# Demographics and histopathological characteristics of enucleated microphthalmic globes

**DOI:** 10.1038/s41598-022-09261-2

**Published:** 2022-03-28

**Authors:** Hind M. Alkatan, Khalid M. Bedaiwi, Yasser H. Al-Faky, Azza M. Y. Maktabi

**Affiliations:** 1 Ophthalmology Department, College of Medicine, King Saud University, Riyadh, Saudi Arabia; 2 King Saud University Medical City, King Saud University, Riyadh, Saudi Arabia; 3 Pathology and Laboratory Medicine Department, College of Medicine, King Saud University, Riyadh, Saudi Arabia; 4 Pathology and Laboratory Medicine Department, King Khaled Eye Specialist Hospital, Riyadh, Saudi Arabia

**Keywords:** Consanguinity, Eye abnormalities

## Abstract

Microphthalmia is a rare ocular anomaly with a poorly understood etiology that is most likely related to heritable and/or environmental factors. Many papers have been published pertaining to the clinical manifestations and management of this condition; however, few reports have reported detailed histopathological findings, which are the focus of this study, in addition to highlighting the basic demographics in these cases. This was a retrospective, observational study of all consecutive enucleated microphthalmic globes (with or without cysts) at 2 tertiary eye hospitals in Riyadh, Saudi Arabia. Globes were classified into 2 groups: severe microphthalmos (axial length or mean diameter less than 10 mm in infancy or 12 mm after age 1 year) and mild microphthalmos based on larger measurements. Clinical and demographic data collected included sex, age at enucleation, eye involvement, nationality/region, consanguinity, family history of eye anomaly, pregnancy, systemic disease, or syndromes. For histopathological data, a descriptive analysis was mostly performed. For correlations of some of our qualitative data, Fisher’s exact test was used. Eleven cases (6 mild and 5 severe microphthalmos) were initially identified with a female to male ratio of 4:7. Ten patients were Saudis, 7 of whom were from the central region. Consanguinity was found in 36% (4/11), and 3 of them had other ocular or systemic abnormalities (duodenal atresia, microcephaly, kidney agenesis, cryptophthalmos, and dysmorphic facial features). Histopathological data were available for 10 cases, half of which showed a coloboma and/or anterior segment anomaly. There was no significant correlation among gender, severity of microphthalmos or the presence of coloboma, although severe microphthalmic globes had a higher median of abnormal intraocular structures (9-interquartile range = 2 compared to 6-interquartile range = 1 in the mild group). Aphakia was found in half of the globes with associated anterior segment dysgenesis. We have concluded that microphthalmos is a visually disabling congenital anomaly that can be isolated or associated with other periocular or systemic anomalies, possibly in relation to consanguinity in our cases. Congenital aphakia was found in half of these cases and was mostly associated with absent Descemet’s membrane and agenesis of anterior chamber angle structures, supporting previously suggested embryological concepts. These findings necessitate further wider genetic testing and proper premarital counseling in Saudi Arabia.

## Introduction

Microphthalmia is a rare congenital birth disease^1^ that can be either unilateral or asymmetrically bilateral. It might also occur as an isolated disease or associated with other congenital or syndromic anomalies^2,3^.

The birth prevalence of microphthalmos and anophthalmos combined reaches up to 30 per 100,000 population. Microphthalmia alone has been reported in up to 11% of children who are legally blind^4^.

The etiology of microphthalmia is not well understood, although epidemiological studies suggest that microphthalmia can be caused by heritable causes, environmental factors, and maternal excessive alcohol intake, in which microphthalmos is one of the manifestations of fetal alcohol syndrome^5^. Mauri and others have reported that the most common environmental cause is gestational-acquired infections such as rubella, toxoplasmosis, varicella, and cytomegalovirus. Additionally, other noninfectious causes, such as vitamin A deficiency, several drugs and exposure to radiation, may be attributed to microphthalmic eyes^2^. Several studies have been published in relation to the epidemiology, clinical spectrum, genetics, and management of microphthalmos with special attention on **m**icrophthalmos, **a**nophthalmos, and **c**oloboma, which are grouped together as MACs^6–9^. However, few individual case reports have focused on the wide histopathological spectrum of features that might be found in association with microphthalmos.

Eye development starts at approximately the 3rd to 10th week of gestation^10^ with optic vesicle formation and then the invagination of the vesicles and formation of the optic cup^11^. In vertebrate eyes, eye development is influenced by interactions between the distal part of the optic vesicles and its contact with the surface ectoderm, which will give rise to the formation of the lens placode. Moreover, invagination of the distal optic vesicle results in the formation of the optic cup with the inner layer giving rise to the future neural retina, while the retinal pigment epithelium develops from the outer layer of the cup^11^. Disruption of this embryogenesis as well as failure of formation of optic vesicles can lead to multiple ocular congenital anomalies^11,12^. The migrating waves of the neural crest from the edge of the neural tube are involved in anterior segment development, and similarly, specific interactions as well as the principles of induction and influence of one developing ocular structure on the growth of another are involved and mediated by epithelial and/or mesenchymal transcription factors, which are the basis behind the genetic background of eye anomalies^13^.

In general, microphthalmia is defined as the presence of any small or hypoplastic globe with a corneal diameter of less than 11 mm or an axial length less than 19 at birth in a newborn and axial length less than 21 mm in an adult^8^. Others have classified microphthalmos into mild (17–21 mm), moderate (12–16 mm), and severe (less than 12 mm) based on the axial length^9^.

In this study, we aimed to determine any association between demographic information and the histopathological characteristics of microphthalmos. We aimed to determine the coexistence of microphthalmos, coloboma, and anterior segment dysgenesis. Identifying these associations would help us plan for future specific genetic studies, providing premarital counseling and possible in utero gene therapy.

## Methods

A retrospective, observational study of all consecutive microphthalmic globes that were enucleated at 2 tertiary eye hospitals: King Khalid Eye Specialist Hospital and King Abdulaziz University Hospital from January 2000 to mid-2021.

All cases were examined by certified ophthalmologists and underwent ultrasonographic and/or computed tomography scanning and magnetic resonance imaging. Because of the generally low prevalence of this condition and the relatively small number of cases, we classified microphthalmos into 2 categories only: the first group was severe microphthalmos with an axial length (or mean diameter) of less than 10 mm in infancy or 12 mm after the first year of age and the second group was mild microphthalmos with measurements of more than 10 mm in infancy or more than 12 mm after the first year of age. The mean diameter of the 3 measured dimensions of the globe was used to classify the cases where the axial length was not specified (to differentiate it from the horizontal and vertical dimensions) by gross examination. The other microphthalmic globes with larger axial lengths (or mean diameter) were grouped as mild microphthalmos.

The collected clinical and demographic data included sex, age at surgical intervention, eye involvement, nationality/region, consanguinity, family history of eye anomaly, pregnancy and delivery, systemic disease or syndromes, ultrasound and/or imaging globe measurements, and visual acuity at presentation. We initially collected 11 cases of microphthalmic globes, 3 of which had cysts. One patient had bilateral microphthalmos with cysts and underwent excision of a large disfiguring cyst on one side without enucleation. This case was included in the demographic basic data, the simple classification into mild versus severe microphthalmos, and the presence or absence of coloboma but excluded from further analysis because of lack of histopathological information.

All histopathological slides were reviewed by 2 experienced pathologists to identify all abnormalities and confirm the presence or absence of coloboma and individual abnormal intraocular structures. These were counted per globe to determine the average number of abnormal structures per 2 groups of mild and severe microphthalmos.

### Statistical analysis

Data were collected on a data sheet form and then transferred to an Excel spreadsheet (Microsoft office 2010). For quantitative data, we used the median. Descriptive analysis was mostly used. For correlations of qualitative data, Fisher’s exact test was used to analyze some of our results. A *p* value of 0.05 was considered statistically significant.

The study was conducted in accordance with relevant guidelines/regulations and in accordance with the Declaration of Helsinki. The research project was granted expedited approval as a retrospective type of study by the Human Ethics Committee/Institutional Review Board (HEC/IRB) at King Khaled Eye Specialist Hospital on 19th May 2021, with an approved collaborative agreement with King Abdulaziz University Hospital (RP-21039-R). Written general informed consent was obtained from all participants and/or their legal guardians for anonymous use of their data. This research does not involve drug trials or human transplantation.

### Ethics approval and consent to participate

This study was approved by the Human Ethics Committee/Institutional Review Board (HEC/IRB) at King Khaled Eye Specialist Hospital with expedited approval on 19th May 2021 as a retrospective study in addition to an approved collaborative agreement with King Abdulaziz University Hospital (RP-21039-R). General informed written consent was obtained from all patients and/or guardians as part of the common practice at both centers.

### Consent to publish

Written consent was obtained from all patients and/or guardians for anonymous use of the data for the purpose of publication.

## Results

### Demographics

A total of 11 cases were initially collected. Only two patients had bilateral microphthalmia (with cysts in one or both eyes), and 9 patients had unilateral microphthalmic globes. Seven out of the 11 cases were males, and 4 were females with a female to male ratio of 4:7. We had 10 Saudi patients and one patient from Yemen. The Saudi patients were mostly from the central region in 7/10. Ten patients were born from full-term uneventful pregnancies, and one was born prematurely at 33 weeks of gestation. Mothers denied relevant drug history or alcohol intake during pregnancy. Consanguinity was confirmed in 4/11 cases (36%), one of whom had bilateral microphthalmos, and the remaining 3 had unilateral microphthalmos.

### Clinical data

Interestingly, 3/4 of these cases who were products of consanguineous marriages (75%) had other associated congenital ocular or systemic anomalies in addition to the microphthalmic globe(s): duodenal atresia and microcephaly in one (Case 2), kidney agenesis in Case 8, and cryptophthalmos with dysmorphic facial features in the third case (Case 11). Six cases of mild microphthalmia and 5 with severe microphthalmia were included (Table 1). Statistically significant associations were not observed between gender and microphthalmos severity (*p* = 0.088). Three cases from each category had posterior coloboma without a statistically significant correlation between gender and the presence of coloboma (*p* ≥ 0.999*).* Similarly, the severity of microphthalmos did not have a significant association with the presence of coloboma (*p* = 0.545). The indication for enucleation in 10 cases was to improve the cosmetic appearance and none of these eyes had undergone previous ocular surgery. One patient underwent surgical excision of a disfiguring cyst without enucleation. Enucleation was performed during infancy in 7 cases, early childhood in 2 cases (at 3 and 4 years of age), and adulthood in 2 patients owing to their delayed presentation.

**Table 1:** Distribution of 11 cases of microphthalmos according to gender, severity of microphthalmos and presence of coloboma (with cyst) with correlation between gender and severity of microphthalmos/presence of coloboma and correlation between severity of microphthalmos and coloboma.

Gender	Mild microphthalmos	Severe microphthalmos	Coloboma with cyst	Total
Male	5	2	4	7
Female	1	3	2	4
Total cases	6	5	6	11

Characteristic		Severity of microphthalmos		P*
		Mild	Severe	
Gender	Male	5	2	0.088
	Female	1	3	

Characteristic		Presence of coloboma		P*
		Yes	No	
Gender	Male	4	3	> 0.999
	Female	2	2	

Characteristic		Severity of microphthalmos		P*
		Mild	Severe	
Presence of coloboma	Yes	2	4	0.545
	No	4	1	

*Fisher’s exact test of association.

### Histopathological findings

**Figure 1:**
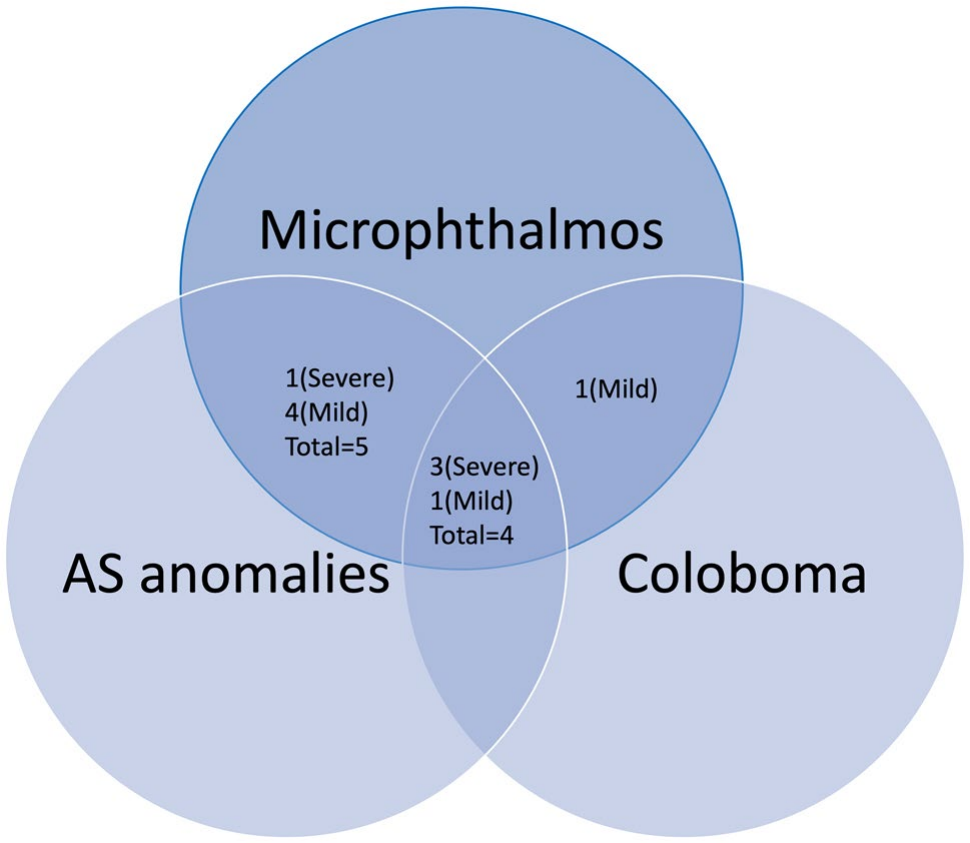
Venn diagrams representing the associations of 10 enucleated globes with microphthalmos, coloboma, and anterior segment (AS) abnormalities in relation to the severity of microphthalmos.

**Figure 2:**
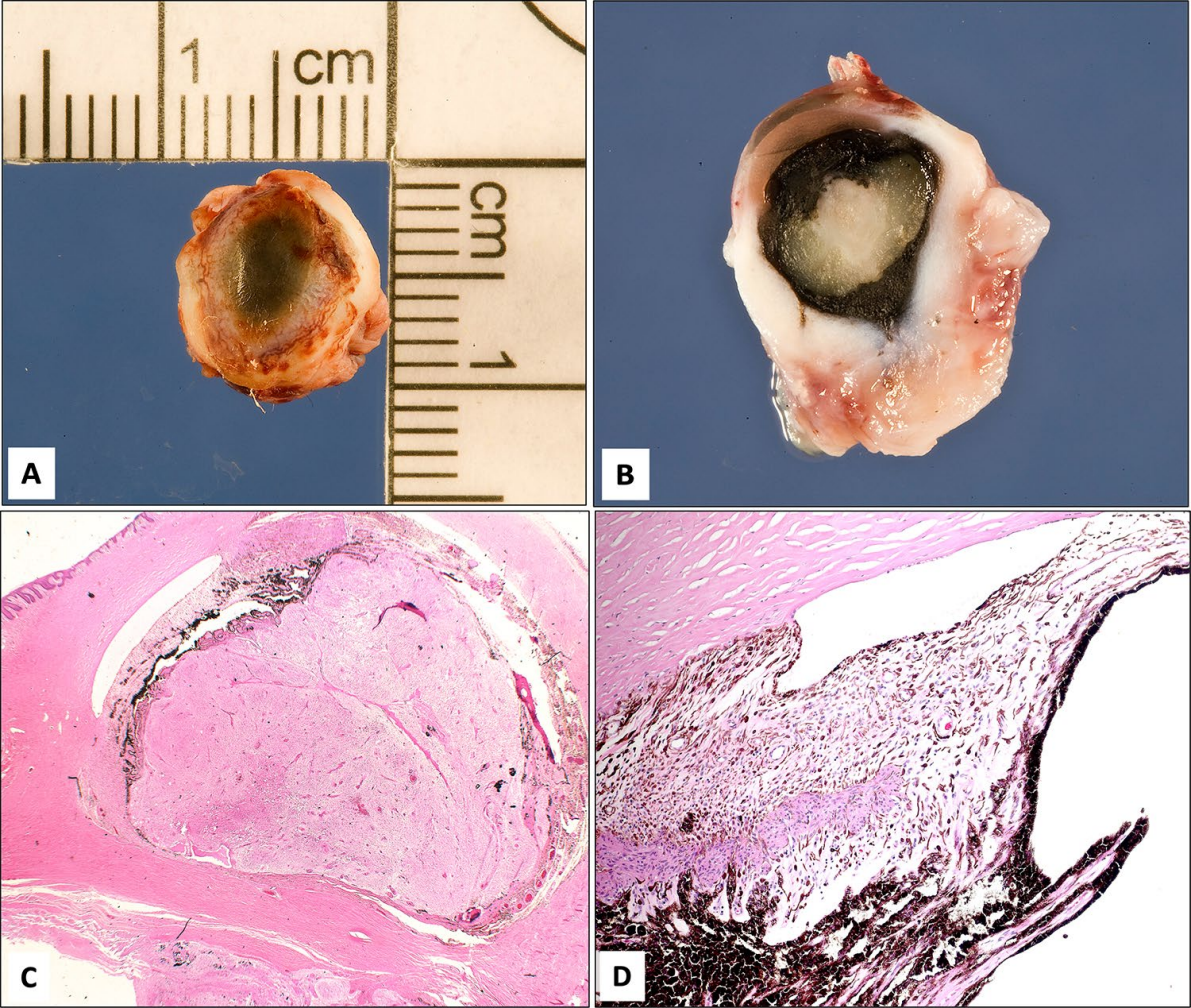
(**A,B**) The gross appearance of a globe with severe microphthalmos. (**C**) Histopathological appearance of the globe with absent lens and dysgenesis of the anterior chamber angle structures and gliosis of the retina (original magnification × 12.5, hematoxylin and eosin). (**D**) Higher power of the anterior segment showing absent angle structures with uveal tissue adherent to the back of the cornea and obliterated narrow anterior chamber (original magnification × 100, hematoxylin and eosin).

The histopathological findings are summarized in Table 2. The median of abnormal intraocular (IO) structures in the mild microphthalmos group was calculated to be 6 (interquartile range = 1), compared to 9 (interquartile range = 2) in the severe microphthalmos group. The overlapping histopathological abnormalities between microphthalmos and anterior segment (AS) anomalies versus coloboma, or both, are shown in Fig. 1. Six cases had posterior coloboma; 5 were chorioretinal in location with combined optic nerve coloboma in 3 eyes (cases 1, 6 and 11), while one eye had optic nerve coloboma with extra-ocular choristoma (case 9). Five patients had an absent lens, and in 3/5, the corneal posterior stroma was abnormal with absent Descemet’s membrane and endothelium. In addition, anterior chamber (AC) angle structures did not develop at all in these 3 cases, while AC dysgenesis was histopathologically observed in the remaining 2 cases (Fig. 2).

**Table 2:** Histopathological characteristics in 10 globes from 11 patients who are diagnosed with microphthalmos.

Case #/Sex	Cornea	AC angle	Iris/CB	Lens	Retina	RPE	Choroid	ON	Diameters mean	Histopathology Dx
1 M*	NA	NA	NA	NA	NA	NA	NA	NA	(By Imaging) Axial length: 7.8 mm	Severe Microphthalmia with Cyst OD (Coloboma). Globe itself was not enucleated
2 M	Thin epithelium/absent BL/stromal NV/absent DM	Agenesis	Hypoplastic, adherent to cornea (leukoma)	Absent	Scarring, hypoplastic, dysplasia	Interrupted focally	Chorio-retinal scarring	Hypoplasia	15 × 14 × 13 Mean: 14 mm	Mild microphthalmia OS
3 F	Irregular epithelium/BL calcification	Dysgenesis	Normal	Absent	Gliosis, hypoplastic	Atrophy	Dystrophic	Absent	18 × 17 × 17 Mean: 17.3 mm	Mild microphthalmia OD
4 M	Irregular BL/thick stroma	Obliterated	Adherent to lens capsule, dilated BV, no crypts	Calcified, spherical	Dysplasia, RD, subretinal fluid	Intact, Drusen-like (few)	Thin, dilated vascular channels	Mild hypoplasia	14 × 11 Mean: 12 mm	Mild microphthalmia OS
5 F	Absent epithelium and BL/scarred stroma/absent DM	Agenesis with adherent CB	Hypoplastic	Absent (aphakia)	Scarring, gliosis, cystic changes, pigment migration	Calcification	Thick, pigment clumping	Absent	12 × 8 Mean: 10 mm	Severe microphthalmia OD with cyst: medullary epithelium, and neuroglial tissue (Coloboma)
6 F	Absent BL (mostly)/scarred stroma/detached DM with attenuated endothelium	Dysgenesis	Hypoplastic	Absent (aphakia)	Hypoplasia, gliosis	Atrophy, focal hyperplasia	Thick	Coloboma, hypoplasia	12 × 9 × 8 Mean 9.7 mm	Severe microphthalmia OD with ON coloboma, IO heterotopic vascular mass and adipose tissue
7 M	Absent BL/thick stroma	Obliterated	Hypoplastic	Clear	Dysplasia	Calcification	Focal lymphocytic infiltrate, dilated vascular channels	Absent	23 × 18 Mean: 21 mm	Mild microphthalmia OD with cyst: glial tissue and fibrofatty tissue. (Coloboma)
8 F	Absent BL/thick scarred stroma/thin detached DM with absent endothelium	Obliterated	Thick, adherent to cornea	Cataract, liquefaction of lens material	Gliosis	Distorted hyperplasia	Distorted with hyperplasia	Absent	9 × 5 × 4 Mean 6 mm	Severe microphthalmia OS
9 M	Not identified	Dysgenesis	Hypoplastic	Resorbed calcified	Dysplasia	Focally present	Pigment clumping	Absent	16 × 10 × 6 Mean: 10.7 mm	Severe Microphthalmia OD with ON coloboma and extraocular choristoma
10 M	Absent epithelium and BL/sclerocornea and NV/partially intact DM with attenuated endothelium	Dysgenesis	Normal	Resorbed calcified	Dysplasia, gliosis	Hyperplasia	Dilated vascular channels	Absent	15 × 10 × 7 Mean: 10.7 mm	Mild microphthalmia OS
11 M	Absent epithelium and BL/stroma NV/absent DM	Agenesis	No iris/CB medullary epithelium hyperplasia	Absent	Dysplasia, gliosis	Intact	Dilated vascular channels and prominent nerves	Hypoplasia, coloboma	10 × 10 × 5 Mean: 12.5	Mild Microphthalmia OD with massive gliosis and ON Coloboma

*M* male, *F* female, *NA* not applicable, *BL* Bowman’s layer, *NV* neovascularization, *DM* Descemet’s membrane, *AC* anterior chamber, *CB* ciliary body, *BV* blood vessels, *RD* retinal detachment, *RPE* retina pigmented epithelium, *ON* optic nerve, *OD* right eye, *OS* left eye, *IO* intraocular.

*Case 1: The globe was not enucleated because it was mildly microphthalmic with clinically normal anterior segment, but the globe had posterior coloboma with a cyst, which was excised. Therefore, histopathological ocular details were available for 10 globes.

**Figure 3:**
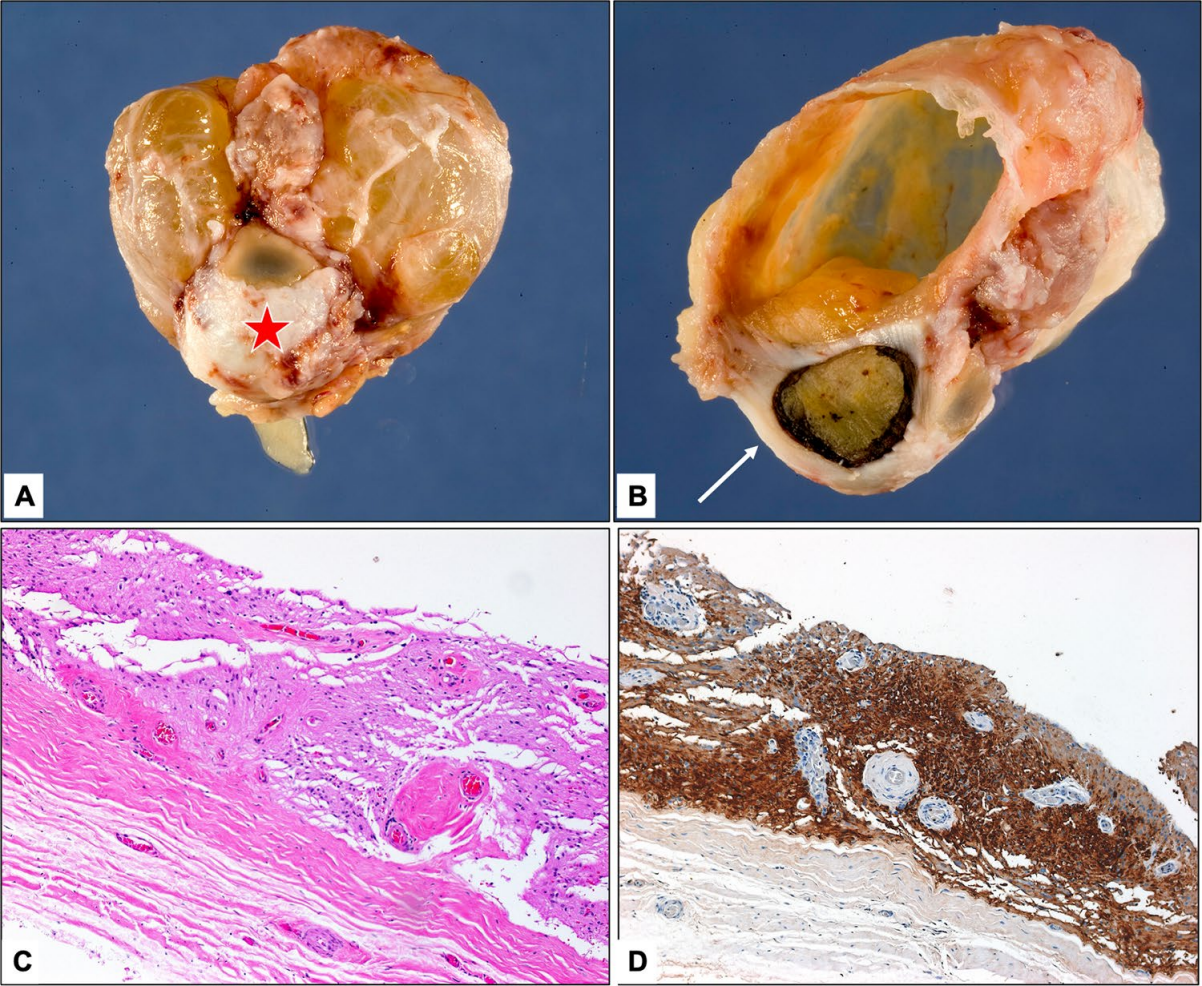
(**A,B**) An example of severe microphthalmos (globe is marked with a red star in (**A**) and a white arrow after sectioning in (**B**)) associated with a large cyst. (**C**) The histopathological neuroglial lining of the cyst wall (original magnification × 100, hematoxylin and eosin). (**D**) Staining of the wall with glial fibrillary acidic protein (GFAP) (original magnification × 100, GFAP).

The associated cysts in 3 cases with coloboma (Cases 1, 5, and 7) were all lined by neuroglial tissue, with medullary columnar epithelium in 2 and fibrofatty tissue in 2 (Fig. 3).

**Figure 4:**
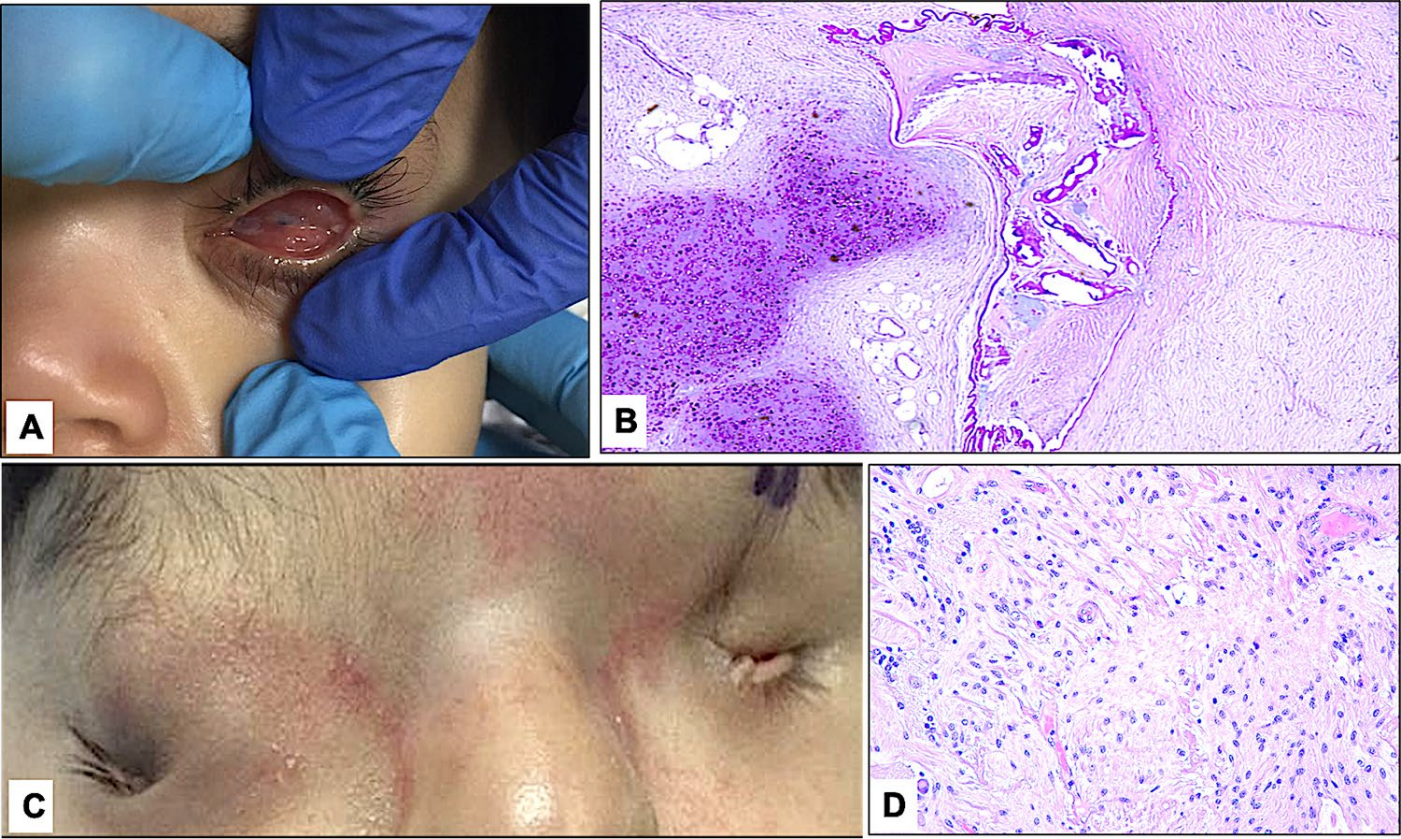
(**A**) The clinical appearance of the left microphthalmic globe in case 10. (**B**) The corresponding histopathological appearance of the incidental intraocular choristoma with cartilage and fat in addition to the calcified lens with wrinkled capsule in that globe (original magnification × 50, periodic acid Schiff). (**C**) The clinical appearance of a dysmorphic periocular area with bilateral cryptophthalmos and microphthalmic left eye in case 11. (**D**) The histopathological appearance of the retina in the enucleated globe with evidence of massive gliosis (original magnification × 200, hematoxylin and eosin).

Interestingly, we had one case of extraocular choristoma (case 9), one with malformed lids and IO choristoma (case 10), and the third showing cryptophthalmos, with massive gliosis of the retina (case 11). Representative histopathological photos for these unique cases are shown in Fig. 4.

## Discussion

Anophthalmia and microphthalmia are rare congenital disorders caused by disruption of the ocular developmental process resulting in an absent or small malformed globe^14^. It has an overall prevalence (combined) in the United States of America (USA) of 3.0 per 10,000 live births. Because of the rarity of this condition, studies on the exact prevalence of this birth defect are not available; however, in a large eye tertiary care center, microphthalmia was observed with a frequency of 36.5 patients per year^8^. This condition might be present as an isolated anomaly alone or can be associated with other global structural defects, such as coloboma; therefore, microphthalmia, anophthalmia, and coloboma have often been grouped together as MAC^15,16^. In a prospective study by Shah and coauthors in the United Kingdom (UK), the MAC cumulative incidence by the age of 16 years was 11.9 per 100,000, with geographical variation observed among Scotland, England, and Wales and racial variations, with a higher risk among Pakistani ethnicities^15^.

Regarding the pathogenesis, little is known about the exact etiology of microphthalmos. It has been suggested that a lower socioeconomic status might carry a higher risk of MAC, and a higher prevalence of isolated anophthalmia/microphthalmia was also found in mothers with a history of fetal deaths and diabetes^14,15^. In our study, patients were born to healthy mothers with uneventful pregnancies, and most of the patients were from the central region of the country, which is mostly urban in nature. In the USA, 55.7% of cases were syndromic or showed chromosomal anomalies, while in nonsyndromic patients, at least one more birth anomaly was found in most cases (92.4%). In our country, we tend to have more isolated cases in 73.2%, while the rest have shown systemic involvement mainly affecting the central nervous system^8^ based on clinical examinations and systemic evaluations of patients to rule out any syndromic features or other associated anomalies without genetic testing. Similarly, in our study, 8/11 patients had isolated microphthalmia (73%).

Regarding bilaterality, the results have not been consistent among different studies, with more than half of the bilateral cases (55.5%) being reported in the UK compared to 41.6% previously reported in the Middle East and 27% only in our current study^8,15^. Chaudhry and his coauthors also studied microphthalmic globes with cysts and reported bilateral involvement in 26% of their cases^9^. It is important to note that his study and ours might not represent the actual frequency of bilateral cases since the study population was peculiar in each of them being limited to microphthalmos with cysts in the first study and limited to enucleated globes in the second. It has also been observed that associated systemic involvement was more commonly seen in bilateral cases^8^.

The issue of common consanguineous marriages in Saudi culture was raised in the same local study by Galindo and her coauthors, where parental consanguinity was found in almost half of the patients with microphthalmia/anophthalmia (46.7%); however, the authors did not correlate the presence of other systemic anomalies to consanguinity^8^. We also detected consanguineous marriage of the parents in 36% (4/11), and 3 out of these 4 patients had associated variable systemic and/or ocular birth defects. On the other hand, none of our 7 cases who were products of non-consanguineous marriages had an associated systemic birth defect and one patient had only ocular malformed lids.

Clinically, microphthalmia can also be associated with congenital cysts, which are another rare congenital developmental defect that develops in the early neonatal period resulting from failure of embryonic fissure closure^9^. The condition can be clinically misdiagnosed as encephalocele, dermoid cyst, arachnoid cyst, or tumors and often requires orbital imaging to reach the correct diagnosis^9,17^. Microphthalmia with cysts can also be associated with posterior segment coloboma and severely malformed eyes. It can be attached as a small appendage to the globe or can be large enough to push the globe and obscure the appearance of a visible globe, thus resulting in the suspicion of anophthalmia clinically because of a hidden small globe^17^. We had 6 cases of coloboma, and half were accompanied by the presence of a cyst. Although the presence of a cyst is expected to be associated with severely malformed globes, the presence of coloboma was not associated with the microphthalmos degree (mild versus severe) or sex. Chaudhry and others have studied the histopathological features of cysts and found that cysts are mainly composed of outer collagenous connective tissue with inner neuroectodermal tissue^9^. Lieb and his coauthors in their case report nicely demonstrated the microscopic, immunohistochemical (IHC) staining and electron microscopic findings of the microphthalmos-associated cyst^18^. They found retinal dysplastic tissue and glial tissue and demonstrated IHC staining with glial fibrillary acidic protein^18^. In our study, all cysts showed similar linings with additional components in 2 consisting of columnar epithelium and fibrofatty tissue in addition to neuroglial tissue with similar IHC staining properties (Fig. 3). Other variably interesting histopathological findings have also been observed in our microphthalmic globes, including IO choristoma and massive gliosis (Fig. 4); however, these results have been reported in detail and will not be further emphasized in our study^19,20^.

Most of the published data on anophthalmos/microphthalmos with or without cysts focus on the clinical aspects, management options, and outcome where the indications for surgery were mostly cosmetic and depended on the size of the globe and/or the cyst in addition to the patient’s condition^8,9^.

It is well known that neural crest cells are essential for the development of the corneal endothelium, corneal stroma, and iris stroma successively, with the theory that the formation of a functional corneal endothelium is essential for the proper formation of the AC^21–23^. It has been found that extracting the lens from chick embryos at early stages resulted in a lack of differentiation of neural crest cell aggregates; thus, the corneal endothelium was not properly developed, and the corneal collagenous architecture was abnormal, resulting in opaque scleral-like stroma^24^. In our cases, 5 had aphakia (absent lens) and in all these cases, the AC angle structures were either not formed at all or showed faulty development (3 cases of agenesis and 2 with AS dysgenesis).

Prokudin and his coauthors have demonstrated the genetically based relation between the coexistence of anophthalmos/microphthalmos, coloboma/or retinal abnormality, and AS abnormalities/cataract/microcornea/Peter’s anomaly^16^. This further proves the complexity of ocular maldevelopment conditions with overlapping phenotypes, as observed in our cases. Posteriorly, all our cases had some sort of abnormality (dysplasia and/or gliosis) of the retina. Generally, none of our cases showed simple microphthalmos without other ocular anomalies, and even the single case with the normal lens was associated with coloboma, as illustrated in Fig. 1. Therefore, microphthalmos should not be considered an isolated anomaly, and the genetic heterogeneity behind its presence should always be taken into consideration. Mutation of different encoding gene proteins can lead to multiple different eye developmental defects, such as microphthalmia/anophthalmia, AS dysgenesis/cataract/microcornea, Peter’s anomaly and coloboma/retinal anomalies^16^. These structural defects can be inherited and follow the Mendelian mode of inheritance pattern or can be sporadic^16^. This wide heterogeneity in gene mutations in relation to microphthalmos makes it challenging to test for genes in all these cases. The overlap between the genes and clinical features of eye maldevelopment makes it difficult to categorize, and a better understanding of eye development is a prerequisite for evaluating and managing complex congenital eye anomalies, especially when associated with systemic disorders and/or chromosomal abnormalities^25^.

The limitations of our study are the small sample size owing to the rarity of microphthalmos, variability of the management options for the condition with a limited number of enucleated globes, and the lack of genetic testing for our cases. Nevertheless, we have concluded interesting observations by extensively studying the details of the histopathological findings in these enucleated globes in correlation with the clinical information.

## Conclusions

In conclusion, several studies have shown that there is an association among microphthalmia, coloboma and anterior segment/cornea abnormalities. Microphthalmos is still considered rare in this part of the world and mostly unilateral and isolated. Cases with a history of parental consanguinity are more likely to have other associated ocular and systemic anomalies necessitating systemic evaluation by pediatric geneticists. Sex did not correlate significantly with the degree of microphthalmos or the presence of coloboma. Severe microphthalmos was observed to be associated with a higher average number of IO abnormal structures but not necessarily with coloboma. We observed that the absence of the lens was associated in most cases with AS and/or corneal abnormalities, thus supporting the overlapping steps in ocular development and the complexity of the involved molecular genetics. This observation necessitates solid family counseling, especially for consanguineous couples, a proper pathway for pediatric referrals and the availability of genetic testing whenever needed.

## Supplementary Information

Supplementary Information.

## Data Availability

The data used in this study are submitted as Supplementary Material.
